# Treatment of Typhoid Fever in the Philadelphia Hospitals

**Published:** 1880-11-20

**Authors:** 


### THE TREATMENT OF TYPHOID FEVER IN THE PHILADELPHIA HOSPITALS.

*The Hospital of the University of Pennsylvania.*—The remedies which have been found at the University Hospital to exert the most powerful influence upon the follicular intestinal catarrh, always present in this disease, are first and foremost the nitrate of silver, and next the subnitrate of bismuth and carbolic acid. There would seem to be abundant evidence that nitrate of silver reduces the size of the enlarged follicles, relieves the inflammatory engorgement, and allays the hyperæthesia of the nerves. It has also been settled by numerous experiments that the nitrate of silver is the most easily administered of the three astringents above mentioned, and the best tolerated by the system. If there is any putrid element in the disease, carbolic acid is employed instead of the nitrate of silver. The nitrate of silver is administered in doses of onefourth of a grain, four times a day. This treatment is persevered in until the ulcers have entirely healed.

If the discharge from the bowels is composed of small, semi-solid stools, it is, with propriety, disregarded; but if the stools are watery and large, opium is administered in pill-form, combined with the nitrate of silver. From one-quarter to one grain of the powdered opium is given three times a day. If there is constipation instead of diarrhœa, belladonna is given conjointly with the nitrate of silver.

Great care is had with regard to the diet when the catarrhal inflammation of the intestines is present. The food employed is, of course, as digestible as possible. Milk has been found to be the best diet in this disease. If the curd appears in the stools, the milk is diluted with water, or lime-water. Of this mixture of milk and lime-water three ounces are given every two hours, or a little over two pints in the course of the twenty-four hours. When the bowels are torpid, beef or mutton broth is given alternately with the milk.

The beef-tea employed is prepared after the following recipe: Take a quantity of tender meat, and, after cutting off the fat, chop it up fine, put it in a bowl, pour a pint of water over it, and let it stand over-night. The water should be kept just on a simmer—the temperature never being allowed to go above 140°, otherwise all the albumen is coagulated, and so either left on the sieve in straining, or introduced into the stomach in the form of curds. After this simmering solution has been allowed to stand over-night, pour it into a pipkin, and heat it again gently with enough salt to give it flavor, and, if necessary, add a drop or two of muriatic acid. Then pour it out over a hair-sieve into a jar. The resulting solution will be found to contain all the nutriment possible, and to be the most valuable kind of stimulant and laxative.

When the fever is high, the patient is given all the food he can take. Care is had, however, that, in allowing food, the already inflamed intestinal tract is not further irritated.

The poison in the blood is controlled by means of quinia, and nitro-muriatic or salicylic acid. As a general thing, salicylic acid is only employed where there is some putrid discharge joined with high fever. Quinia is considered (1) to neutralize the effects of the septic poison in the blood, (2) to act as a good tonic to the muscular and nervous systems, (3) to tend to check febrile action, and (4) to remove any malarial element that happens to be present. Quinia is never given in the enormous doses advised by the German physicians. It has been found that such doses will break down high fever, but they produce entirely unnecessary irritation of the gastric mucous membrane. About twelve grains of quinia are given in the course of the twenty-four hours.

The temperature is kept down by preventive measures rather than by the cold bath, which is regarded as a last resort. It is unnecessary after this to say that the practice of the University Hospital is wholly opposed to the indiscriminate cold bathing in typhoid fever, so much in vogue in Germany within a year past.

When the temperature runs up in spite of drugs, in the milder cases, spongings of the whole body are practiced every two hours, the sponges being squeezed out of a mixture of water and bay rum at a temperature from 60° to 80°. If this does not succeed (it rarely fails), and if the patient's temperature mounts up to 104° or 105°, he is then wrapped up in sheets wrung out of cold water. If the temperature still runs up to such an extent that life is threatened, the patient is placed in a cool bath until the bodily temperature is sufficiently reduced.

Before the local lesions appear, the fever can be more boldly attacked; but when, in subsequent stages, it runs high, it is regarded as partaking of the nature of a sympathetic fever, largely dependent upon the amount of intestinal lesion, and the use of baths at this period is thought to be attended with great risk. If the cold bath is used at all (except as a last resort, and when temperature cannot be reduced in any other way), it is employed during the first ten days in cases where the temperature rises above 103° and cannot be controlled by frequent spongings, large doses of quinia, diaphoretics, etc.

With regard to the use of stimulants, the hospital practice is not in favor of administering them simply because the patient has the fever. It is believed that stimulants are only demanded for the relief of certain symptoms. As a general thing, they are not given to children before the age of puberty. They are only administered to old persons, and to meet certain indications, viz., (1) ataxic nervous disturbances, such as sleeplessness, twitchings of the muscles, maniacal delirium; (2) circulatory disturbances, such as feeble and rapid pulse, and feeble development of the first sound of the heart; (3) profound asthenia, as shown by great tremulousness, inability to make any movement, and tendency to slide down off the pillow; (4) dry and brown tongue, with sordes on lips, teeth, and tongue.

The milder forms of stimulus are always used at first. The one most frequently employed is wine-whey. This is made in the proportion of one part of sherry to three of milk, and as much as a gill or half a pint of it given in the course of three hours. If the symptoms increase, stronger stimulants are used, such as whiskey. Whiskey is usually given in lime-water and milk; the lime-water prevents the coagulation of the milk by the alcohol These ingredients are mixed in the proportion of one tablespoonful each of whiskey and lime-water to every three ounces of milk. In this form half an ounce of whiskey is given every hour. If the stimulation is doing good, a diminution of the serious symptoms is noted. If the symptoms increase, on the other hand, the amount of stimulus is reduced.

With regard to complications: relapses are always regarded as true second attacks of the disease, and are treated accordingly. The treatment is resumed, the diet restricted, and the same general watchfulness had over the state of the case as during the course of the first attack.

Hemorrhage occurring early in the attack is considered as of but little consequence, but when it supervenes later—when the sloughs are thrown off—it is regarded as a very serious matter. The treatment of hemorrhage is by absolute rest in bed for twenty-four hours, and by the administration of opium, to produce complete quiet for the alimentary canal. The opium is given by the rectum, one grain of the solid opium being prescribed every two or three hours until the patient is gently under its influence; of astringents, for local action, acetate of lead is preferred. A suppository containing one grain of opium and three grains of the acetate of lead is given three or four times daily. Ergot, by reason of its action upon the walls of the arterioles, is also very highly prized. It is given hypodermically near the supposed seat of the hemorrhage. The food allowed is very small in quantity, and absolutely liquid.

Peritonitis is treated by antiphlogistics, sedatives, perfect rest in bed, and a diet which leaves no residuum to irritate the bowels.

True perforation is regarded as beyond the reach of medical skill to mend.

*The German Hospital.*—The quinine treatment (heroic doses) has been given a fair trial in the wards, and has been found to do but very little, if any, good. It has not even been satisfactorily demonstrated that it reduced the temperature, as the same changes in temperature have taken place in the case of those who have been taking the mineral acids alone. Indeed, after giving quinia some time, in some cases it was stopped, and the same changes were found to exist. Quinia has seemed rather to increase the diarrhœa and headache, and in two cases it produced entire deafness for two weeks. Sponging with vinegar and water has been found to act beneficially. Plenty of ice is given the patient to suck, and the ice-cap is applied to the head. The wet pack has been found to lower the temperature for the time being, but in an hour or more it generally mounts up again. To this is added the consideration that it has the disadvantage of necessitating the constant moving of the patient, wearing and weakening the constitution, thereby destroying his or her main support against the disease.

Oil of turpentine, as recommended formerly by Dr. George B. Wood, has been proven to act most beneficially. Especially has it been found useful in those cases where the dry, dark, and heavily coated tongue exists, with abdominal symptoms. It is given in twenty-drop doses in mucilage, every hour or two, and is continued in smaller doses during convalescence. In a large number of cases in which dry, dark tongue existed with tympanites, turpentine acted most beneficially, the tongue regaining its normal color and becoming moist in from six to eight days, and the tympanites disappearing in a much shorter time.

The mineral acids are of great service in keeping the stomach in good order, stimulating the appetite and relieving the intense thirst. In many cases the patients call for their dose of the acid hours before the time, so much are they pleased with its taste and effects. The acid common- used is the dilute nitro-muriatic acid.

Whenever active, wild delirium exists, from one-third to one-half of a grain of morphia is given hypodermically. This medication has been found to act promptly in almost every instance. In one case particuarly, the patient towards evening showing signs of approaching delirium, a large dose of morphia was immediately given hypodermically, which had the effect of rendering the patient perfectly rational when he awoke. Upon another occasion, when this same patient again showed signs of approaching delirium, the morphia was omitted, upon which a wild attack of delirium came on, which was at once broken up by the use of a moderate dose of morphia hypodermically.

*The Episcopal Hospital.*—The temperature is reduced and the heart strengthened by fifteen-drop doses of the tincture of digitalis and two grains of quinia, every three hours. Stimulants are only employed in the severer cases. Excessive diarrhœa is controlled by injections containing fifteen drops of laudanum and a half a fluid ounce of starch. Dilute muriatic acid is given in fifteen-drop doses every three hours, and in the second week of the disease five drops of turpentine are administered every three hours. Hemorrhage from the bowels is controlled by the internal use of ergot, and the local application of ice to the abdomen. A number of cases have been treated of late with one-fourth grain doses of the nitrate of silver in the second week of the disease, this dose being repeated every three hours with entirely negative results.

*The Pennsylvania Hospital.*—Ten grains of quinia are given daily, and ten drops of muriatic acid every three hours. The patient is sponged all over with cold water, in the mornings and evenings. Diarrhœa is controlled by opiates and astringents. This is the routine treatment. The diet is very carefully regulated, consisting principally of beef-tea and milk. When the first sound of the heart is altered (weakened) early in the course of the disease, it is regarded as an indication that the patient should immediately be put upon the use of stimulants; or, if he is already taking whiskey, that the daily amount should be doubled.—*Medical Record, N. Y.*

